# Objective improvement with coronary anastomosis simulation training: meta-analysis

**DOI:** 10.1093/bjsopen/zrab147

**Published:** 2022-01-28

**Authors:** Marliza O’Dwyer, Cristina A. Fleming, Shane Ahern, Sean Barrett, Nicola B. Raftery, Tara Ní Dhonnchú, Kishore Doddakula

**Affiliations:** 1 Department of Cardiothoracic Surgery, Cork University Hospital, Cork, Ireland; 2 Department of Academic Surgery, Cork University Hospital, Cork, Ireland; 3 Department of Breast Surgery, Mater Misericordiae University Hospital, Dublin, Ireland

## Abstract

**Background:**

Coronary artery anastomosis training and assessment are vital for patient safety and for conferring a prognostic benefit. A systematic review and meta-analysis were performed to analyse the impact of simulation on coronary anastomosis proficiency in terms of time taken and skill score.

**Methods:**

This review was conducted in accordance with PRISMA guidelines, searching PubMed, Embase and Cochrane databases on 10 October 2020, using the terms ‘Coronary anastomosis simulation’ or ‘vascular anastomosis simulation’ and ‘anastomosis simulation’. Studies included had objective measurement of scores of before and after simulation. Meta-analysis was performed using RevMan, version 5.4 (Cochrane Library).

**Results:**

From a pool of 1687 articles, 12 articles evaluating the use of simulation in teaching coronary anastomosis were identified, with objective scores at baseline and after simulation. The 12 papers included 274 subjects. Data on 223 subjects could be extracted for analysis in performing coronary anastomosis in a simulated environment. Eight trials evaluated improvement in time and 12 trials evaluated performance using an objective evaluation score. In comparison with no formal simulation training, simulation was associated with improved skill in a five-point scale (standardized mean difference 1.68 (95 per cent c.i. 1.23 to 2.13; *P* < 0.001)) and time (mean difference 205.9 s (95 per cent c.i. 133.62 to 278.18; *P* < 0.001)) in trials included in the meta-analysis. Furthermore, novice cardiothoracic surgeons benefited more from simulation as regards time improvement compared with senior cardiothoracic surgeons (293 *versus* 120 s improvement; *P* = 0.003). Fidelity of simulator did not have a significant effect on rates of improvement.

**Conclusion:**

Simulation-based training in coronary anastomosis is associated with improved time efficiency and overall performance in comparison with no intervention. Further studies are necessary to determine the optimum timing of trainees progressing from simulation training to live operating.

## Teaser

Summary of findings from this study: 274 subjects performing coronary anastomosis were included in this review. They improved their coronary anastomosis with simulation for both time and skill.

## Introduction

Coronary artery bypass grafting surgery has been the cornerstone of cardiac surgery since its development in 1960 by Goetz and colleagues^1^. Results of the ROOBY (Randomized On/Off Bypass) trial highlight the significance of excellent surgical technique, whether on-pump or off-pump, in improving graft patency, with a 16.4 per cent rate of adverse events at 1 year in patients with ineffective revascularization compared with a 5.9 per cent rate in the effective revascularization group^2^. Therefore, it is an axiom that patient safety be at the forefront of educating trainees in performing coronary anastomosis. This paradigm has shifted medical-education models from apprenticeship towards simulation training^3^.

Approaches to simulation in educating trainees in cardiothoracic procedures appears to offer appropriate training without risk to patients or trainees^3–19^. It has been employed widely and has shown efficacy in the education for adult cardiac surgery, thoracic surgery, transcatheter aortic valve implantation, robotics, perfusion, cardiac advanced life support and extracorporeal membrane oxygenation^12^. Training for coronary artery bypass grafting differs amongst consultant trainers and training programmes. Reviewing the evidence with respect to the effectiveness and key steps of coronary anastomosis simulation would permit training bodies to target problem areas and identify remaining research needs.

No previous meta-analysis or review has been published regarding how objective scores and times improve with simulation in coronary artery bypass grafting. The aim of this study was to identify, analyse and summarize comparative studies objectively assessing coronary anastomosis simulation by conducting a systematic review of the literature.

## Methods

A systematic review and meta-analysis of coronary artery anastomosis performance comparing simulation to no prior simulation was performed in adherence to PRISMA standards of quality for reporting meta-analysis^20^. The specific aim was to analyse if simulation could improve either objective competency scores or time to task completion. No ethical approval was required for this study.

### Eligibility

Studies involving healthcare trainees at any stage in training that were evaluated while using simulation to teach coronary anastomosis in comparison with no intervention (that is, a control arm or preintervention assessment) were included. Single-group pre-test/post-test and two-group randomized and non-randomized trials were included. Exclusion criteria were articles not available in English and those which did not refer to coronary anastomosis simulation training. Studies reporting on health professionals using biological tissue and synthetic material to perform coronary anastomosis in simulation in comparison to no simulation were included.

### Study identification

PubMed, Embase and Cochrane databases were searched on 10 October 2020, using the terms ‘Coronary anastomosis simulation’ or ‘vascular anastomosis simulation’ and ‘anastomosis simulation’. Two independent investigators participated in study selection and data abstraction was performed independently and in duplicate.

### Study selection

Study selection involved two stages. In stage 1, all studies evaluating coronary artery simulation were identified. Full-text versions of all publications that could not be excluded with confidence were obtained and reviewed for definite inclusion, independently and in duplicate using the Covidence platform. In stage 2, studies in which an end-to-side vascular anastomosis were performed with the goal of simulating coronary anastomosis, and those in which time and/or objective score improvement were assessed objectively were identified. End-to-end anastomosis simulation or large vessel anastomosis or studies in which coronary anastomosis was not addressed were excluded. When conflict arose, the paper was discussed between two reviewers to clarify inclusion and exclusion criteria. No paper required a third reviewer.

### Data extraction

Information was abstracted separately for time improvement and Objective Structured Assessment of Technical skill (OSAT) score improvement. OSAT score improvement was further subclassified into individual segments of arteriotomy, graft orientation, bite/depth of bite, spacing, use of needle holder, use of forceps, needle angles, needle transfer, suture management, knot tying, hand mechanics, use of both hands and economy of time if provided in the text. If surgical skill score or time score was not reported, it was not included in the meta-analysis.

### Data synthesis

Studies were grouped according to the comparison arm, that is scores before simulation and those after simulation. To analyse scores homogeneously, scores and standard deviations were converted to a five-point scale, where 1 corresponded with a poor score and 5 corresponded with an excellent score. For quantitative synthesis, results were pooled using meta-analysis, comparing time to completion (in seconds) and five-point score before simulation with time to completion (in seconds) and five-point score after simulation. Qualitative synthesis of trials was also included, identifying curriculum design features, to assess salient themes.

### Risk of bias

The Cochrane Risk of Bias tool and the Newcastle–Ottawa quality assessment scale (NOS) for non-randomized cohort studies were applied to determine objectively the quality of each eligible study^21,22^. The Cochrane tool considered bias under the following headings: selection bias, performance bias, detection bias, attrition bias, reporting bias or other bias^22^. For the NOS, points were awarded for patient selection (maximum four points), outcome assessment (maximum three points) and comparability of cohort (maximum two points): each added to a maximum of nine points^21^.

### Statistical analysis

Competency scores and time to complete tasks were reported on continuous scales. Mean and standard deviation were extracted and calculated for continuous outcomes. The mean difference was the difference in competency score or time to complete a task before and after simulation. Objective scores were inverted or multiplied as required to match other five-point Likert scales. Owing to inherent differences in study populations and clinical sites, random effects meta-analyses were conducted for all outcomes. The weighted mean differences or standard mean differences with 95 per cent confidence intervals were calculated for continuous variables using the inverse variance method. The Cochrane chi-squared and *I^2^* statistic were used to examine the heterogeneity among effect estimates in included studies. Statistical heterogeneity among studies was defined as *I^2 ^*> 50 per cent. Review Manager version 5.4 (The Nordic Cochrane Centre, The Cochrane Collaboration, Copenhagen, Denmark) was used to perform the meta-analysis and to generate forest plots^23^. *P* < 0.050 was considered statistically significant.

## Results

### Literature search

**Fig. 1:**
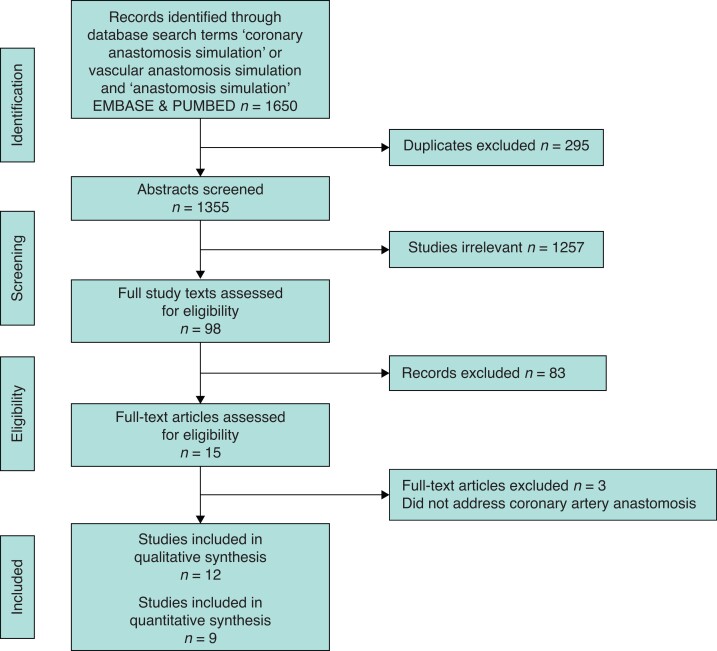
PRISMA flow diagram

The search strategy identified 1650 articles, demonstrated in *Fig. 1*. After eliminating duplicates and selecting out studies evaluating simulation in coronary artery anastomosis, 12 articles were identified. These studies evaluated simulation-based coronary anastomosis training in comparison with no intervention and these were included in the meta-analysis and are summarized in *Table 1*. Out of eleven studies which evaluated an objective score improvement, seven could be used in quantitative meta-analysis. Furthermore, the OSAT scores of each of the steps in coronary anastomosis, if available, were subanalysed in these score-improvement studies. Out of eight studies which evaluated time improvement, six could be used in quantitative meta-analysis. All articles were published in English. None were identified in any other language. Study design is described in *Table 1*. Risk of bias assessment is summarized in *Tables 2* and *3*. The majority of outcome data was fully available, ensuring a lack of attrition bias between studies. Heterogeneity between study designs introduced selection and selective reporting bias.

**Table 1:** Characteristics of included studies

Study and year	Design	Population sample	Endpoints evaluated	Comparison	Fidelity of simulator	Time spent in study	Inter-rater reliability of examiners
**Anand *et al*., 2020^7^**	Prospective, observational	17 (6 senior and 11 junior)	Thoracic Surgery Directors Association Vessel Anastomosis Assessment Score improvement (13 technical categories using a five-point Likert Scale)* Time improvement use of simulator and attitudes survey	Baseline and quarterly scores over 1 year Improvement in junior *versus* senior residents	Low-fidelity cardiac simulator Chamberlain group coronary anastomosis pocket simulator	1 year	Unclear
**Wu *et al*., 2020^8^**	Blinded, prospective trial	60	Performance of anastomosis evaluated according to a five-point global rating scale†	Performance of anastomosis was evaluated at the beginning (after 1 month), the midpoint (after 2 months), and the end of the assessment (after 3 months). Compared beating and non-beating heart simulator scores	High fidelity. Porcine hearts and remnants of human saphenous veins were used as grafts for anastomoses. To simulate the coronary artery bypass under the condition of a beating heart, an intra-aortic balloon in the left ventricle was used	3 months	>0.65, demonstrating moderate reliability
**Spratt *et al*., 2018^14^**	Prospective randomized study	12 (senior)	Blinded skill assessments were captured by video at 0, 8 and 16 weeks using the Joint Council on Thoracic Surgery Education Assessment tool (out of a score of 50).* Survey of simulation use	16-week curriculum *versus* control operative experience Tests before and after simulation in control and treatment trainees	Low-fidelity cardiac simulator (Synaptic Design, Minneapolis, MN)	16 weeks	Single blinded grader
**Malas *et al*., 2018^9^**	Single-blinded randomized prospective trial	32 (junior)	Objective Structured Assessment of Technical Skill (OSATS) scale (max score 25)† Anastomosis-specific end-product rating score Time to completion	Control group: performed simulation at home Treatment group: received additional instructional multimedia to use independently	Low fidelity (Limbs & Things, Savannah, Georgia, USA) and non-ringed, 4-mm polytetrafluoroethylene grafts (W. L. Gore & Associates, Inc, Flagstaff, Arizona, USA)	1 week	2 blinded expert observers
**Tavlasoglu *et al*., 2015^10^**	Single-blinded randomized prospective trial	15 (junior and senior)	Time-improvement assessment of the anastomoses were evaluated with binary existence of anastomotic leak, additional suture requirements, matching between graft diameter and arteriotomy length, patency rates and inadvertent posterior wall injuries	First, second and third month of study sequential assessments	High fidelity Bovine simulator	3 months	3 blinded cardiac surgeons
**Maluf *et al*., 2015^15^**	Prospective trial	10 mixed	Sequential tests after simulation * Time improvement*	Compared serial improvements using different simulators	Low-fidelity and high-fidelity simulators: Arroyo box simulator Sim model with dummy Sim model with bovine heart Sim model with pulsatile porcine heart	6 months	Not available
**Enter *et al*., 2015 Top Gun^16^**	Prospective trial	17 junior	Modified OSATS, included 12 component skills scored on a five-point Likert scale Time improvement Questionnaire on demographics, prior surgical experience and simulation	Video assessment of anastomosis of 17 residents at baseline *versus* anastomosis of 15 residents after simulation	Low-fidelity simulator (Chamberlain Group, Great Barrington, Massachusetts, USA)	6-week simulation practice	Robust
**Enter *et al*., Med Students 2015^17^**	Prospective single-blinded, randomized controlled trial	45 junior	Tests before and after simulation using a five-point Likert scale (JCTSE Assessment Tool) Self-evaluation performance scores Interest in Surgery survey	Control (n = 15) no practice *versus* unsupervised (n = 15) *versus* supervised (n = 15)	Low-fidelity simulator (Limbs & Things, Savannah, Georgia, USA)	4 weeks	Blinded expert raters
**Nesbitt *et al*., 2013^18^**	Blinded, prospective, randomized intervention trial	21 participants (10 junior and 11 senior)	Modified OSATS using a five-point scale but reported using interquartile range* Time to completion*	Medical students who underwent simulation training *versus* senior residents with no simulation training No baseline scores reported in the text	Porcine simulator	4 months	Fair to moderate
**Ito *et al*., 2012^19^**	Blinded, prospective, intervention trial	4 senior	Each resident performed 40 anastomoses In total, 160 anastomoses were done with comparison of baseline and final anastomosis performed using time to completion and five-point scale of five components up to a maximum of 25 points†	Baseline first 10 anastomoses compared with final 10 anastomoses	Low-fidelity BEAT, YOU-CAN simulator	2 months	Fair to moderate
**Fann *et al*., 2010^11^**	Blinded, prospective, intervention trial	33 first-year cardiothoracic surgical residents	Global rating scale for assessment of coronary anastomosis based on a one-to-three-point model† Mean performance rating scores were also used to evaluate aspects of anastomosis Exit survey to assess perception of coronary artery simulator	At beginning, midpoint, and session end, anastomosis components were compared on a three-point rating scale (1 good, 2 average, 3 below average)	Low-fidelity portable High-fidelity explanted pig hearts, expired cryopreserved saphenous veins (Cryolife, Inc, Kennesaw, Georgia, USA)	2.5-day boot camp	>0.5
**Fann *et al*., 2008^5^**	Blinded, prospective, intervention trial	8 senior	Tests on each model before and after simulation Time improvement Five-point objective performance rating scores†	Compared outcomes on beating heart model and non-beating with tests before and after simulation	High-fidelity beating-heart model with silicone beating-heart model (Chamberlain Group, Great Barrington, Massachusetts, USA) using 3-mm silicone vein grafts	1 week	Good reliability

* Unable to use data for analysis. †This was reversed or adjusted for the purposes of analysis.

**Table 2:** Result of the critical appraisal using the Newcastle–Ottawa Scale

Study and year Design Score out of 9	Selection				Comparability	Outcome		
	Representative sample	Selection of non-exposed cohort (no simulation)	Ascertainment of exposure	Demonstration of training score before simulation	Based on design of analysis Comparability/reproducibility of cases and curriculum	Assessment of outcome	Curriculum length reported	Adequacy of follow-up
**Anand *et al*., 2020^7^** **Prospective** **observational** **Score = 8**	+	−	+	+	++	+	+	+
**Wu *et al*., 2020^8^** **Single-blinded prospective randomized trial** **Score = 7**	+	−	+	−	++	+	+	+
**Spratt *et al*., 2018^14^** **Multicentre prospective** **randomized trial** **Score = 6**	+	+	+	+	–	+	+	–
**Malas *et al*., 2018^9^** **Single-centre, single-blinded randomized prospective trial** **Score = 7**	+	−	+	+	++	+	+	+
**Tavlasoglu *et al*., 2015^10^** **Single-blinded randomized prospective trial** **Score = 6**	+	−	+	−	+−	+	+	+
**Maluf *et al*., 2015^15^** **Single-centre prospective trial** **Score = 3**	+	−	+	−	–	−	+	−
**Enter *et al*., 2015 Top Gun^16^** **Multicentre prospective trial** **Score = 7**	+	−	+	+	++	+	+	+
**Enter *et al*., 2015 Med Students^17^** **Prospective single-blinded, randomized controlled trial** **Score = 8**	+	−	+	+	++	+	+	+
**Nesbitt *et al*., 2013^18^** **Single-centre, single-blinded, prospective, randomized intervention trial** **Score = 6**	+	+	+	−	+	−	+	+
**Ito *et al*., 2012^19^** **Single-centre, single-blinded, prospective, intervention trial** **Score = 8**	−	−	+	+	++	+	+	+
**Fann *et al*., 2010^11^** **Single-centre, single-blinded, prospective, intervention trial** **Score = 8**	+	−	+	+	++	+	+	+
**Fann *et al*., 2008^5^** **Single-centre, single-blinded, prospective, intervention trial** **Score = 8**	+	−	+	+	++	+	+	+

**Table 3:** Cochrane Collaboration’s Risk of Bias assessment

Study and year	Selection bias		Performance bias	Detection bias	Attrition bias	Reporting bias	Other bias
	Random sequence generation	Allocation concealment	Blinding (participants and personnel)	Blinding (outcome assessment)	Incomplete outcome data	Selective reporting bias	Other sources of bias
**Anand *et al*., 2020^7^**	High	High	High	Unclear	Low	Low	Low
**Wu *et al*., 2020^8^**	Unclear	Unclear	Low	Low	Low	Low	Low
**Spratt *et al*., 2018^14^**	Low	Low	Low	Low	High	High	High
**Malas *et al*., 2018^9^**	Low	Low	Low	Low	Low	Low	Low
**Tavlasoglu *et al*., 2015^10^**	Unclear	Unclear	Low	Low	Low	Low	Low
**Maluf *et al*., 2015^15^**	High	High	High	Unclear	High	High	Low
**Enter *et al*., 2015 Top Gun^16^**	High	High	Low	Low	Low	Low	Low
**Enter *et al*., Med Students2015^17^**	Unclear	Unclear	Low	Low	Low	Low	Low
**Nesbitt *et al*., 2013^18^**	Unclear	Unclear	Low	Low	Low	Unclear	Low
**Ito *et al*., 2012^19^**	High	High	Low	Low	Low	Low	Low
**Fann *et al*., 2010^11^**	Unclear	Unclear	Low	Low	Low	Low	Low
**Fann *et al*., 2008^5^**	Unclear	Unclear	Low	Low	Low	Low	Low

### Study characteristics

In total, 223 out of 274 potential participants (178 residents or trainees in surgery and 45 medical students) were assessed in meta-analysis. Further demographics of participants based on age and gender in relation to level of improvement were not reported consistently. No study assessed performance in the context of coronary anastomosis in real patients. The only study which had a control group defined as normal operative intervention was that by Spratt and colleagues^14^.

**Fig. 2:**
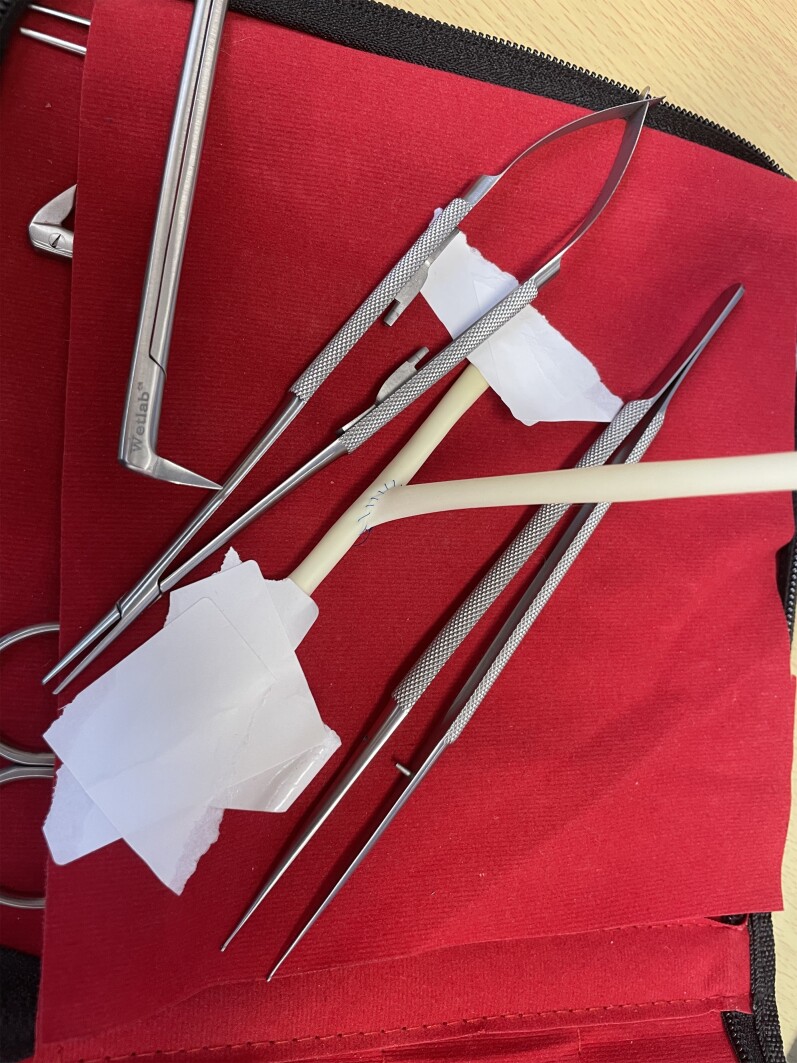
Coronary anastomosis low-fidelity simulator End-to-side anastomosis of Limbs & Things (Savannah, Georgia, USA) 6-mm vein, performed with 6-0 prolene, using Castroviejo needle holder and atraumatic forcep, available from wetlab.co.uk.

Simulation modalities varied amongst trials. These included both low- and high-fidelity simulation models. Low-fidelity models were described as three-dimensional multistation cardiovascular simulators^14^, the Arroyo box Simulator, vessel anastomosis simulators with silicone vein grafts (Chamberlain Group, Great Barrington, Massachusetts, USA; Limbs & Things, Savannah, Georgia, USA) and the ‘BEAT, YOU-CAN’ model^7,9,11,14–17^. Such models are made of a range of synthetic materials with 3-mm silicone ‘vein grafts’ which are suitable for multiple end-to-side anastomoses (*Fig. 2*). The beating heart models are also silicone based and are connected to an external compressor which simulates the movement of the beating heart. High-fidelity models included bovine hearts and pulsatile pig hearts^5,8,10,15,18^. Five studies used biological tissue, while the rest used low-fidelity or a combination of low- and high-fidelity models. The heterogeneity of these models introduced a risk of bias in analysing the data.

An instructional video was provided in three trials^5,14,17^. In person education sessions were provided in four trials^14,15,17,18^. Baseline and follow-up video characteristics were undertaken in four trials^14,16–18^.

Curriculum design was not uniform across the trials, and length of study period or curriculum ranged from 2.5 days to 6 months. One trial mandated a minimum of 20–30 min of dedicated technical practice each week for 8 weeks, however the authors noted that participants may not have committed to this timetable^14^. Maluf and colleagues’ training programme spanned over 6 months using four different models^15^. The Top Gun group undertook 6 weeks of simulation^16^, while Enter and co-workers’ medical students had 1 month of training^17^. Nesbitt’s team evaluated simulation over 4 months^18^. Fann and colleagues gave residents the task station to practice and evaluated improvement after 1 week^5^. There was an over-riding theme of self-directed learning. Only Enter and colleagues, evaluated the impact of supervision on improving simulation scores in medical students. There was no statistically significant difference in supervised simulation and non-supervised simulation.

### Effectiveness in improving time and score in comparison with no formal simulation

**Fig. 3:**
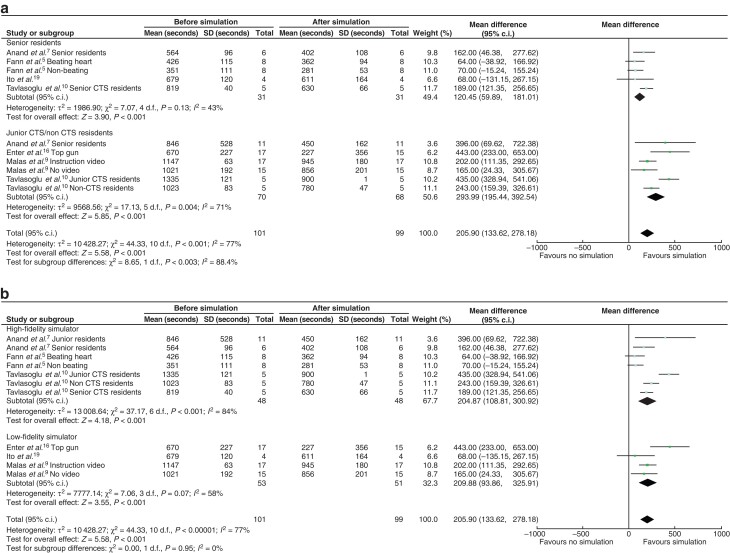
Forest plots comparing before *versus* after simulation in evaluating time improvement (in seconds) Subgroup analysis: **a** comparing senior residents with junior cardiothoracic surgery(CTS)/non-CTS residents; **b** comparing high-fidelity simulator with low-fidelity simulator.

Eight studies evaluated time improvement comparing simulation-based training with presimulation score^5,7,9,10,15,16,18,19^. Data from six of these studies could be included in meta-analysis. Four of these studies^5,7,9,10^. had more than one group, which are listed in *Fig. 3*. For time analysis, there were eleven groups who were assessed prior to and after simulation. A mean difference of 205.9 s was observed (95 per cent c.i. 133.52 to 278.18; *P* < 0.001) in trainees who used simulation (*Fig. 3*). Individual effect sizes ranged from 64 to 443 s. There was also presence of heterogeneity: *I^2^*= 77 per cent. The forest plot favoured simulation training with respect to time.

**Fig. 4:**
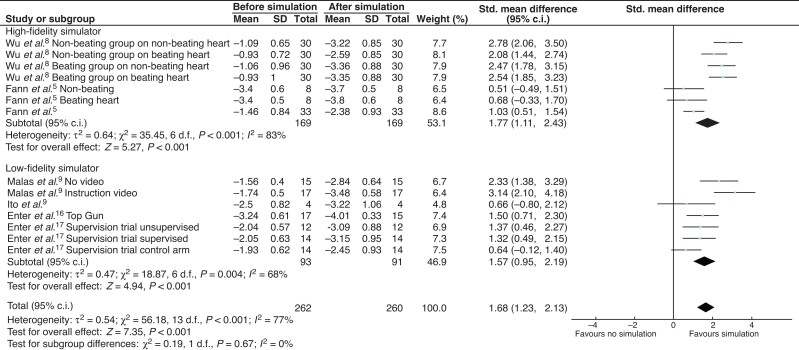
Forest plot comparing no simulation *versus* simulation in evaluating improvement in skill score Skills evaluated using a five-point scale where 0 = poor score and 5 = excellent score. Subgroup analysis comparing high-fidelity simulator with low-fidelity simulator.

Twelve studies evaluated score improvement, comparing simulation-based training with no intervention. Data from seven studies could be included in meta-analysis. Four studies^5,8,9,17^. evaluating score improvement had more than one group which underwent simulation, and these are listed in *Fig. 4*. There were 14 groups assessed prior to and after simulation as regards score improvement. Trainees who used simulation had an associated improvement of 1.68 points (95 per cent c.i. 1.23 to 2.13; *P* < 0.001) (*Fig. 4*). Heterogeneity was present between studies, with *I^2 ^*=* *77 per cent and individual effect sizes ranging from 0.51 to 3.14 points. The forest plot favoured simulation training with respect to score improvement.

Furthermore, the use of simulation in improving OSAT score on specific tasks was analysed (*Fig. S1*). Simulation was associated with significant improvement in all trainee scores in arteriotomy, graft orientation, bite/depth of bite, spacing, use of castro/needle holder, use of forceps, needle angles, needle transfer, suture management, knot tying, hand mechanics, use of both hands, economy of time and configuration of anastomosis. Heterogeneity was present in many of these specific tasks.

### Simulation effect on level of training

Nesbitt and colleagues demonstrated that simulation training improved medical students’ time to completion and skill score with coronary anastomosis to a level comparable to that of residents, while the senior residents had only a non-significant trend towards improvement^18^. Subgroup analysis demonstrated that simulation in junior and non-cardiothoracic residents was associated with more of a positive impact when compared with senior residents, as regards time improvement (293 *versus* 120 s, *P* = 0.003), in a test for subgroup differences (*Fig. 3*). However, the heterogeneity between groups was high and thus limits interpretation of this result. There was no significant difference in time improvement when comparing high- and low-fidelity simulator trials. In subgroup analysis of score improvement between high- and low-fidelity simulators, there was no significant difference between groups in subgroup differences (*P* = 0.67) (*Fig. 4*). Again, heterogeneity scores made this difficult to interpret.

## Discussion

In comparison with no intervention, simulation was consistently associated with better learning outcomes in two broad categories: time and OSAT score improvement. Simulation permits surgical trainees to acquire complex and meticulous skills without jeopardizing patient safety. The results can also be explained anecdotally. The phrase ‘practice makes perfect’ can be demonstrated graphically, plotting performance against experience, which usually produces a sigmoid curve. Repeated application of learned principles through simulation is key to achieving success in transferring skills from the working and sensory memory to the long-term memory.

Issenberg and co-workers described the necessary features for simulation to transform a principle into a lesson learned^24^. This includes provision of feedback, repetitive practice, curriculum integration, appropriate range of difficulty level, multiple learning strategies, simulation that captures clinical variation, a controlled environment, individualized learning, defined outcomes and simulator validity^20^. Simulation, and meta-analysis of simulation, has been carried out in other aspects of cardiothoracic surgery, for example, bronchoscopy^3,12^. Simulation has also shown to be useful in real-life scenarios, with a study by Ledermann and co-workers evaluating junior orthopaedic residents, demonstrating that when trainees use a low-fidelity simulator to perform an arthroscopic partial meniscectomy, their score in the Arthroscopic Surgical Skill Evaluation Tool improved in real-life patients^25^.

When senior trainees utilized coronary artery simulators, it did not appear to have a significant effect in improving scores and performance^14^. A suggested reason for this is that senior trainees have lack of flexibility and time in utilizing simulators due to clinical practice and there may be diminishing marginal benefits from additional practice^7,24^. Anecdotally, the flimsy design of low-fidelity simulators has been a barrier for their use. Novice or junior trainees appeared to benefit from simulation across the trials^17^. The non-significant trend towards improvement noted in Nesbitt and colleagues’ study in senior residents may also be explained by achieving a proficiency, which only needs to be practised occasionally^18^. It should be noted that junior and senior trainees may not be distinguishable after multiple simulations given there may be a plateau or a ceiling effect^5,26^.

Regarding curriculum design, the use of instructional videos and homework plans was explored in these studies^5,14,17^. Furthermore, it was shown that using high-fidelity porcine simulators increased trainee interest in that specialty^11^. Only one study evaluated the effect of additive supervision in anastomosis training and this did not appear to have a significant effect in training^17^. However, this study only included 45 medical students and may well have been insufficiently powered^17^. The present study highlights aspects of current teaching methods and the need to create dynamic curricula that create proficient surgeons^27–29^.

The transition from simulation to real-life patients has not been explored in the literature in coronary artery anastomosis. However, it has been identified that after 30 anastomoses on the simulator, the learning curve stabilized^5,15,19^. Furthermore, in a study of 15 cardiothoracic surgeons by Bridgewater and colleagues, it was identified that newly appointed consultant cardiothoracic surgeons had an improvement in the mortality rate of their low-risk patients undergoing coronary artery bypass graft after 4 years compared with established surgeons^30^. Studies need to evaluate when it is best for a trainee to progress from low-fidelity to high-fidelity simulators and to patients.

Quantity and quality of studies limit the results of this study, along with clinical and methodological diversity. The search criteria and analysis were limited to studies which focused on coronary anastomosis simulation, however there may be merit in including end-to-side anastomosis simulation utilized in vascular surgery, for example, femoral bypass. There were significant differences in approaches between studies. In particular, the quality and amount of instruction given to anastomosis performers in each of the trials were difficult to compare. Verification of simulation practice at home could not be documented accurately. The reality of having a first assistant is not explored in these studies. The length of time and quality of dedicated practice to simulation was not taken into account in the meta-analysis. OSAT scores were similar and inter-observer reliability was maintained in individual studies, but heterogeneity exists between studies. Outcomes of time and score were determined objectively in individual studies, but a significant limitation was a lack of a control group for simulation groups with test scores before and after simulation. Progression with operative experience could account for improved scores, particularly in the lengthier studies. While heterogeneity between studies exists, a positive impact with the use of simulation is demonstrated throughout.

This is the first meta-analysis of the use of simulators in coronary artery anastomosis and it provides a basis for reforming training in cardiac surgery. These data suggest simulation improves steps and time to completion in coronary anastomosis, therefore it is suggested that these steps be focused on during training days and trainees should be encouraged to practise at home. Heterogeneity of studies limited the interpretation of whether simulation is more beneficial for junior trainees than for established surgeons, thus more controlled studies should evaluate when when it is best for trainees to transition from simulation to live operating.

Future research should look at cost analysis of low- and high-fidelity models and consider the provision of these models to surgical trainees. Training bodies could then focus simulation days, taking into account cost and time. Use of faculty time is an important consideration in employing their use. Training bodies should balance the cost of simulation with the inherent benefit of improving interest in the specialty.

Simulation is an accepted form of teaching surgical techniques. Trainers may establish a baseline competence before awarding trainees responsibility in the operating theatre. Demonstrating proficiency in simulation may progress trainees faster rather than the traditional apprenticeship model. Identifying appropriate proficiency levels in which trainees may progress from low- to high-fidelity models and subsequently to patients should be identified with further research. Advanced skills, such as managing unexpected bleeding and utilizing assistance, which has not been evaluated by these simulation studies, may be taught in the apprenticeship model in the operating theatre.

## Supplementary Material

zrab147_Supplementary_Data
