# Supplementary material for: Objective improvement with coronary anastomosis simulation training: meta-analysis
Source: BJS Open. 2022 Jan 28;6(1):zrab147. doi: 10.1093/bjsopen/zrab147 (PMC8830760; doi:10.1093/bjsopen/zrab147)
Supplement: zrab147_Supplementary_Data [file zrab147_supplementary_data.zip › Supplementary_Figure_1.docx]

**Supplemental Figure 1**


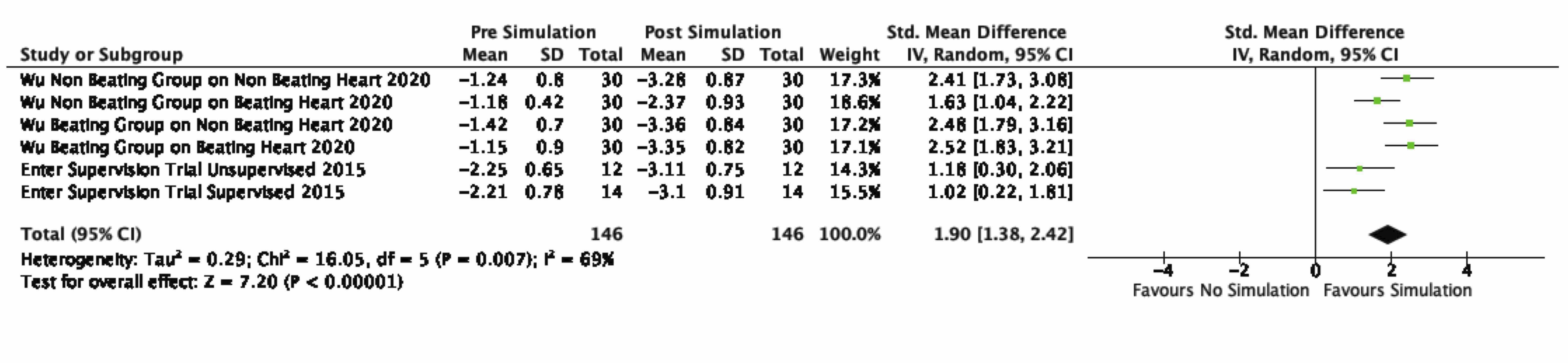


*a: Forest plot of comparison: No Simulation versus Simulation in evaluating Improvement in Skill Score of Arteriotomy in a 5-point scale where 0=poor score and 5=excellent score*

*
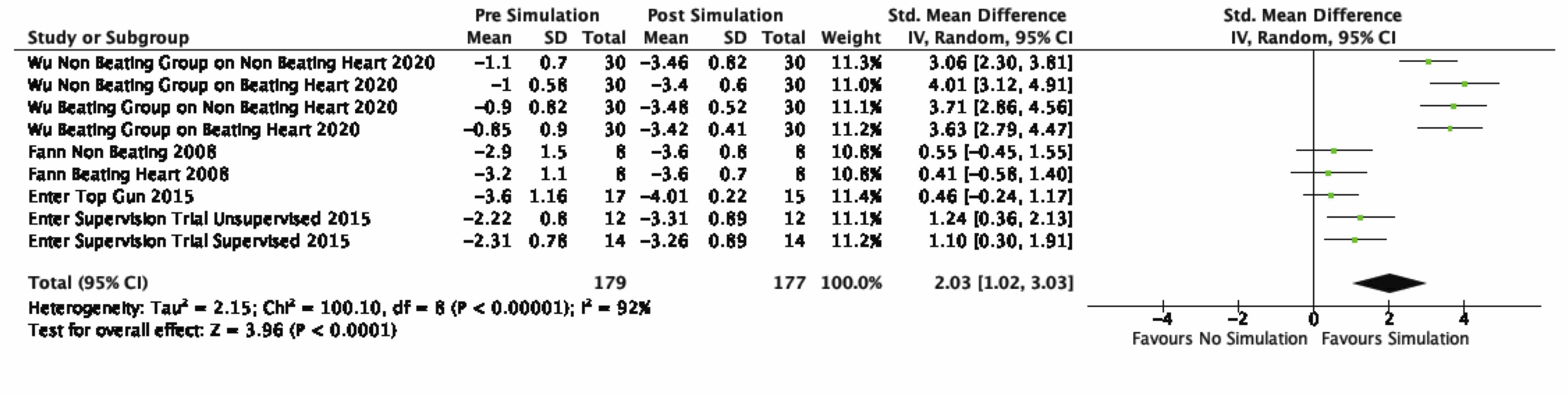
*

*b: Forest plot of comparison: No Simulation versus Simulation in evaluating Improvement in Skill Score in Graft Orientation in a 5-point scale where 0=poor score and 5=excellent score*

*
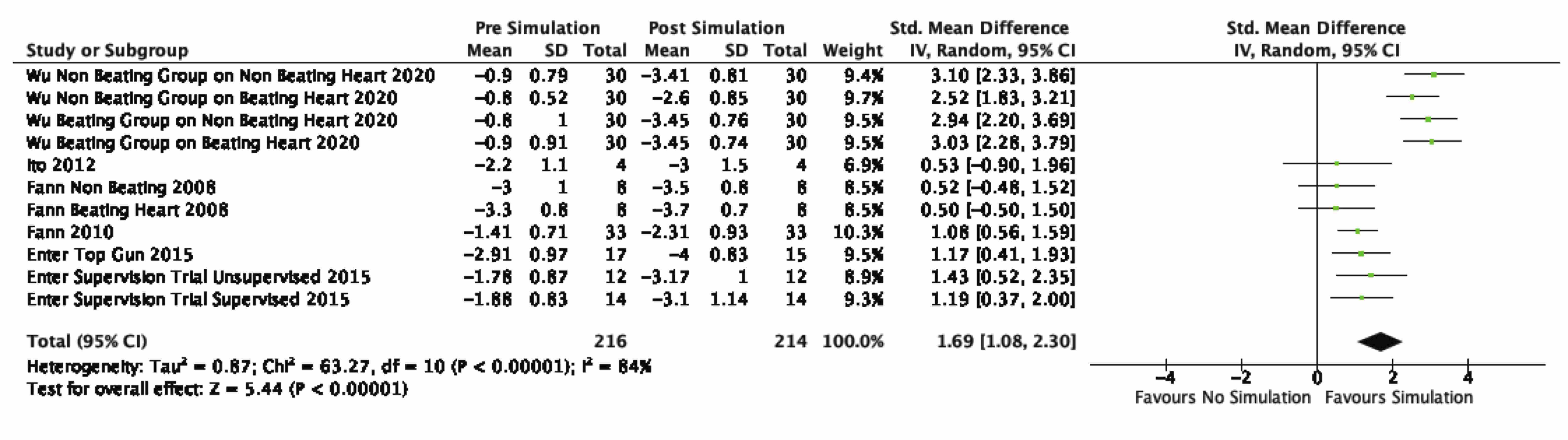
*

*c: Forest plot of comparison: No Simulation versus Simulation in evaluating Improvement in Skill Score in Bite/Depth of Bite in a 5-point scale where 0=poor score and 5=excellent score*

*
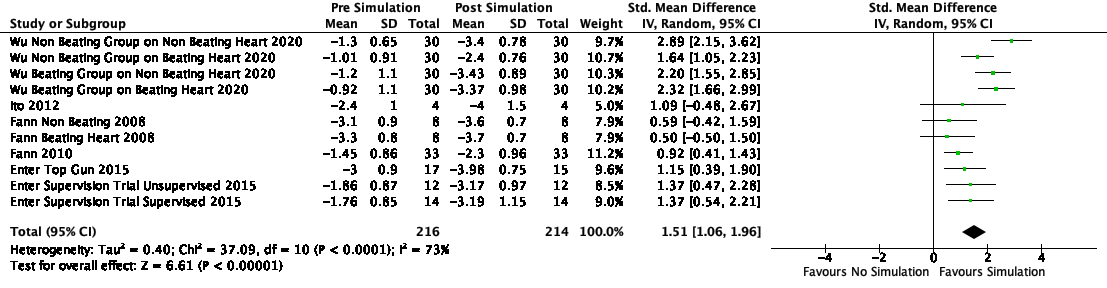
*

*d: Forest plot of comparison: No Simulation versus Simulation in evaluating Improvement in Skill Score in Spacing in a 5-point scale where 0=poor score and 5=excellent score.*

*
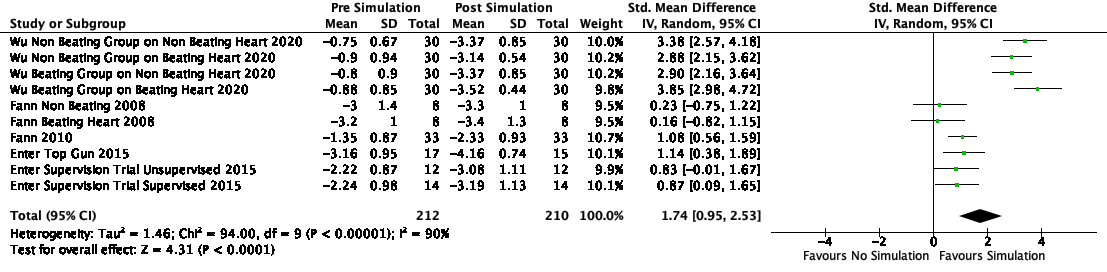
*

*e: Forest plot of comparison: No Simulation versus Simulation in evaluating Improvement in Skill Score in Use of Castro/Needle Holder in a 5-point scale where 0=poor score and 5=excellent score.*

*
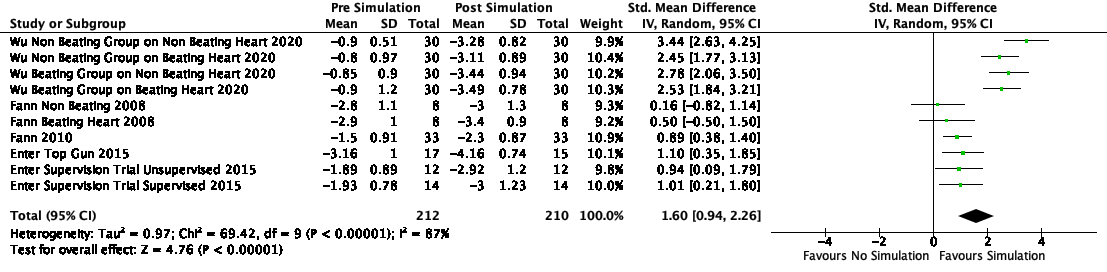
*

*f: Forest plot of comparison: No Simulation versus Simulation in evaluating Improvement in Skill Score in Use of Forceps in a 5-point scale where 0=poor score and 5=excellent score.*

*
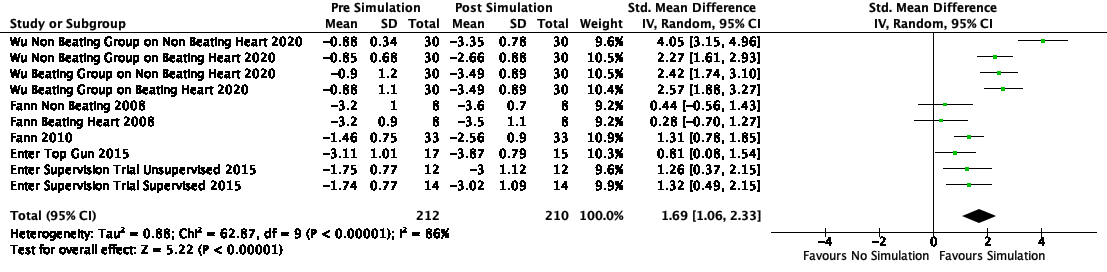
*

*g: Forest plot of comparison: No Simulation versus Simulation in evaluating Improvement in Skill Score in Needle Angles in a 5-point scale where 0=poor score and 5=excellent score.*

*
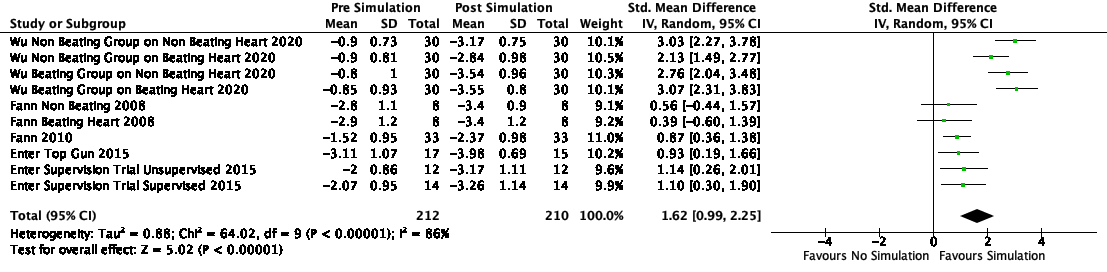
*

*h: Forest plot of comparison: No Simulation versus Simulation in evaluating Improvement in Skill Score in Needle Transfer in a 5-point scale where 0=poor score and 5=excellent score.*

*
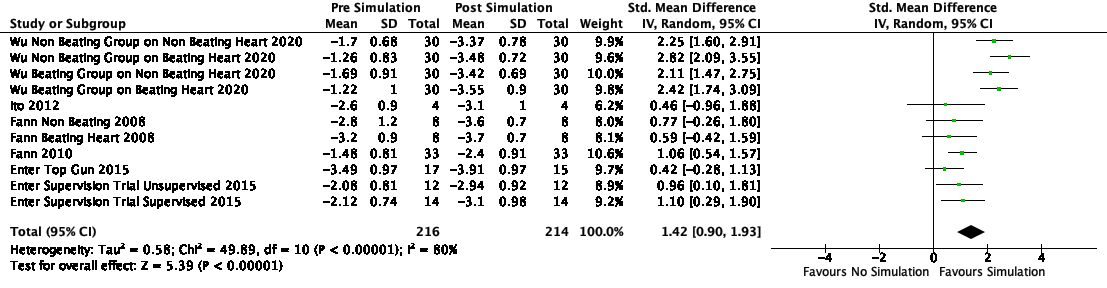
*

*i: Forest plot of comparison: No Simulation versus Simulation in evaluating Improvement in Skill Score in Suture Management in a 5-point scale where 0=poor score and 5=excellent score.*

*
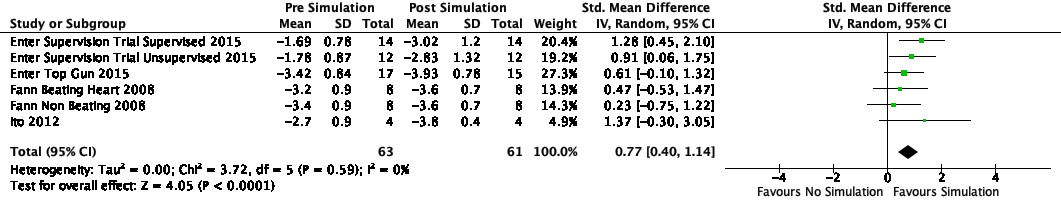
*

*j: Forest plot of comparison: No Simulation versus Simulation in evaluating Improvement in Skill Score in Knot Tying in a 5-point scale where 0=poor score and 5=excellent score*

*
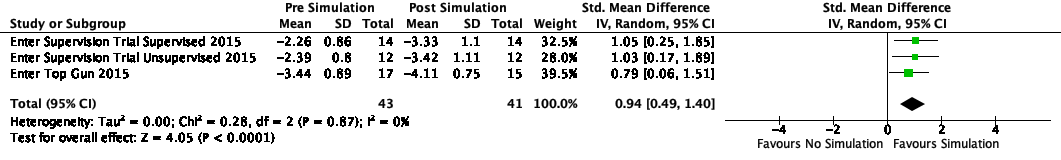
*

*k: Forest plot of comparison: No Simulation versus Simulation in evaluating Improvement in Skill Score in Hand Mechanics in a 5-point scale where 0=poor score and 5=excellent score*

*
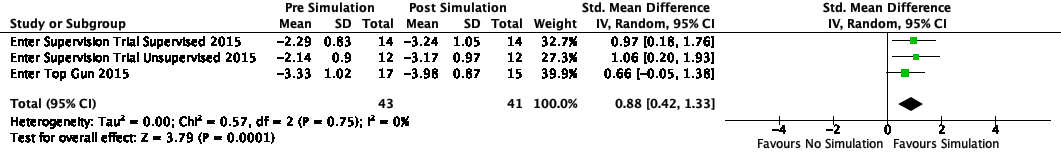
*

*l: Forest plot of comparison: No Simulation versus Simulation in evaluating Improvement in Skill Score in Use of Both Hands in a 5-point scale where 0=poor score and 5=excellent score.*

*
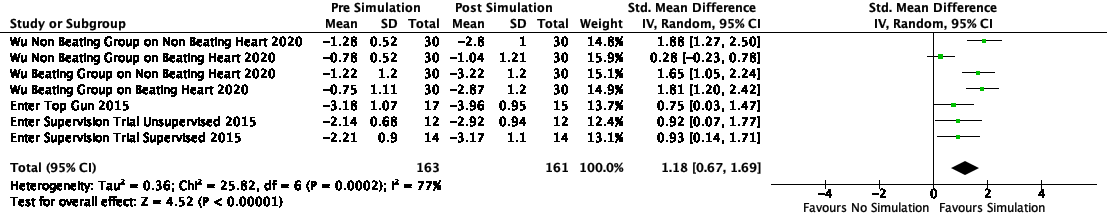
*

*m: Forest plot of comparison: No Simulation versus Simulation in evaluating Improvement in Skill Score in Economy of Time in a 5-point scale where 0=poor score and 5=excellent score.*

*
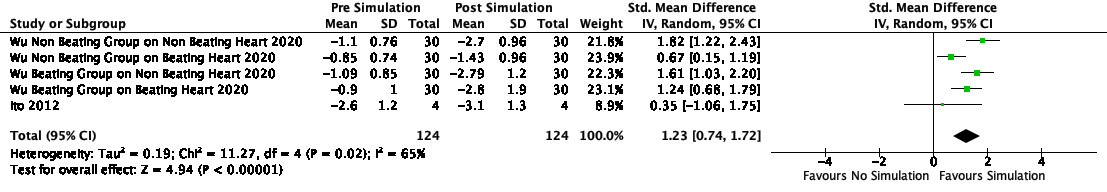
*

*n: Forest plot of comparison: No Simulation versus Simulation in evaluating Improvement in Skill Score in Configuration of Anastomosis in a 5-point scale where 0=poor score and 5=excellent score.*
